# Powerful Bivariate Genome-Wide Association Analyses Suggest the *SOX6* Gene Influencing Both Obesity and Osteoporosis Phenotypes in Males

**DOI:** 10.1371/journal.pone.0006827

**Published:** 2009-08-28

**Authors:** Yao-Zhong Liu, Yu-Fang Pei, Jian-Feng Liu, Fang Yang, Yan Guo, Lei Zhang, Xiao-Gang Liu, Han Yan, Liang Wang, Yin-Ping Zhang, Shawn Levy, Robert R. Recker, Hong-Wen Deng

**Affiliations:** 1 School of Medicine, University of Missouri - Kansas City, Kansas City, Missouri, United States of America; 2 The Key Laboratory of Biomedical Information Engineering of Ministry of Education and Institute of Molecular Genetics, School of Life Science and Technology, Xi'an Jiaotong University, Xi'an, People's Republic of China; 3 Vanderbilt Microarray Shared Resource, Vanderbilt University, Nashville, Tennessee, United States of America; 4 Osteoporosis Research Center, Creighton University, Omaha, Nebraska, United States of America; 5 College of Life Sciences and Bioengineering, Beijing Jiaotong University, Beijing, People's Republic of China; 6 Center of Systematic Biomedical Research, Shanghai University of Science and Technology, Shanghai, People's Republic of China; National Institute of Child Health and Human Development/National Institutes of Health, United States of America

## Abstract

**Background:**

Current genome-wide association studies (GWAS) are normally implemented in a univariate framework and analyze different phenotypes in isolation. This univariate approach ignores the potential genetic correlation between important disease traits. Hence this approach is difficult to detect pleiotropic genes, which may exist for obesity and osteoporosis, two common diseases of major public health importance that are closely correlated genetically.

**Principal Findings:**

To identify such pleiotropic genes and the key mechanistic links between the two diseases, we here performed the first bivariate GWAS of obesity and osteoporosis. We searched for genes underlying co-variation of the obesity phenotype, body mass index (BMI), with the osteoporosis risk phenotype, hip bone mineral density (BMD), scanning ∼380,000 SNPs in 1,000 unrelated homogeneous Caucasians, including 499 males and 501 females. We identified in the male subjects two SNPs in intron 1 of the *SOX6* (SRY-box 6) gene, *rs297325* and *rs4756846*, which were bivariately associated with both BMI and hip BMD, achieving *p* values of 6.82×10^−7^ and 1.47×10^−6^, respectively. The two SNPs ranked at the top in significance for bivariate association with BMI and hip BMD in the male subjects among all the ∼380,000 SNPs examined genome-wide. The two SNPs were replicated in a Framingham Heart Study (FHS) cohort containing 3,355 Caucasians (1,370 males and 1,985 females) from 975 families. In the FHS male subjects, the two SNPs achieved *p* values of 0.03 and 0.02, respectively, for bivariate association with BMI and femoral neck BMD. Interestingly, *SOX6* was previously found to be essential to both cartilage formation/chondrogenesis and obesity-related insulin resistance, suggesting the gene's dual role in both bone and fat.

**Conclusions:**

Our findings, together with the prior biological evidence, suggest the *SOX6* gene's importance in co-regulation of obesity and osteoporosis.

## Introduction

Genome-wide association studies (GWAS) have now become a major strategy for genetic dissection of human complex diseases/traits. Through this strategy, novel genetic polymorphisms have been successfully identified for many common diseases of public health importance. A general trend for current GWAS is to collect multiple phenotypes of interest from a single study population and analyze these phenotypes separately in a univariate framework [1]. However, this strategy is limited by ignoring potential genetic correlation between different traits analyzed and hence is difficult to detect pleiotropic genes that are important to the pathogenesis of many correlated human diseases.

An effective strategy to tackle the challenge of detecting pleiotropic genes is to analyze potentially correlated disease phenotypes simultaneously via a multivariate GWAS approach. This approach takes advantage of covariance between multiple study phenotypes and therefore may be more powerful for detecting pleiotropic genes. In addition, analyzing multiple phenotypes jointly can also alleviate multiple testing problem caused by testing different traits separately. Motivated by the above reasons, we here performed the first bivariate GWAS analyzing simultaneously two correlated diseases of public health significance, obesity and osteoporosis. Our study may set an example for future “multivariate” GWAS of common human diseases.

Obesity is a disease of excessive storage of body fat resulting from chronic imbalance between energy intake and consumption [2]. It is a serious public health problem affecting ∼65% of adult US population [3] and incurring a direct cost of ∼$100 billion per year [4]. Obese people are more likely to develop other serious diseases, such as diabetes, hypertension, and coronary heart diseases [5], [6]. A commonly used measure for quantifying the severity of obesity is body mass index (BMI) that is defined as body weight divided by the square of height.

Osteoporosis is another major public health problem, which is characterized by excessive skeletal fragility and susceptibility to low trauma fractures among the elderly [7]. Currently ∼10 million people in the US suffer from and another ∼34 million are at high risk for the disease [7]. It results in>1.5 million osteoporotic fractures (OF) each year in the US [7] and incurs the country a direct cost of ∼$13.8 billion in 1995 [8]. The most widely accepted measure for quantifying risk of osteoporosis is the amount of bone mass in the skeleton, as denoted by bone mineral density (BMD) [9]. Since hip fracture is the most severe type of OF and directly associated with high morbidity and mortality [10], hip or femoral neck (FN) BMD is the most important risk phenotype for osteoporosis.

Obesity and osteoporosis are closely related diseases [11]. Adipocytes and osteoblasts (the bone formation cell) share the same progenitor, bone marrow mesenchymal stem cells, and can transdifferentiate into each other [12]. Adipocytes secrete factors important to bone remodeling, such as the estrogen synthesis enzyme, aromatase, and proinflammatory cytokines [12]. Increased bone marrow fat was found in osteoporosis patients [13] and correlation between obesity phenotypes (e.g., body weight and BMI) and osteoporosis risk phenotypes (e.g., BMD) was observed [14]–[16]. Several mechanisms were identified in mouse models for fat-bone correlations/interactions and the potential mechanistic links between obesity and osteoporosis, for example, leptin's influence via the sympathetic nerve system [17], [18]. However, the extent to which these mechanisms are relevant to normal human population is still unknown.

Since both obesity and osteoporosis have high genetic predisposition, pleiotropic genes may exist to influence the risks of both diseases, which was supported by studies suggesting significant genetic correlation between the two diseases [11], [19]. Recently, we conducted a bivariate whole genome linkage scan and identified several genomic regions shared by obesity and osteoporosis, providing further support for the existence of pleiotropic genes for the two diseases [20]. Importantly, identification of such pleiotropic genes in humans may offer novel insights into the pathogenic links between obesity and osteoporosis. Such findings, as compared to those from studies using mouse models, may have more direct relevance to normal human population so as to provide important targets for treatment and prevention of both diseases. To identify such pleiotropic genes, we here performed the first bivariate GWAS of obesity and osteoporosis taking advantage of Affymetrix high throughput SNP genotyping platform. Using Affymetrix 500K array, we successfully genotyped and analyzed a total of ∼380,000 SNPs in 1,000 unrelated homogeneous Caucasians. Through bivariate association analyses, we identified the *SOX6* gene (SRY-box 6) as a potential pleiotropic gene underlying both obesity and osteoporosis.

## Results

We compared statistical power of bivariate association analyses of two continuous traits with that of univariate association analyses of each trait separately. According to our power analyses, analyzing two traits simultaneously using bivariate association approach consistently achieved higher statistical power under all the 3 genetic models (i.e., additive, dominant, and recessive) and different SNP effect sizes than analyzing each trait separately using univariate association approach. For example, under the additive model, the model we used in our real data association analysis in this study, the power to detect a QTL of a heritability of 0.01 is under 60% using univariate association analysis approach, as compared with a ∼80% power using bivariate association analysis approach. (A heritability of 0.01 means that the QTL under simulation contributes 1% variation for both traits of a bivariate phenotype, e.g., BMI and BMD.) The detailed results are presented in Appendix S1.

We identified two interesting SNPs in the male subjects of our GWAS cohort, which are *rs297325* and *rs4756846*. The two SNPs, although not univariately associated with BMI or hip BMD at the significance level of *p* = 0.05, were strongly associated with BMI-hip BMD bivariately, achieving *p* values at the levels of 10^−6^ to 10^−7^. The basic characteristics of our GWAS cohort are summarized in Table 1 and the bi/univariate association results are shown in Table 2. In the male subjects of our GWAS cohort, the correlation coefficient between BMI and hip BMD is 0.384 (*p*<0.001), and that between FM and hip BMD is 0.244 (*p*<0.001).

**Table 1:** Basic Characteristics of Study Subjects.

Traits	GWAS cohort		FHS cohort	
	Male (N = 499)	Female (N = 501)	Male (N = 1,370)	Female (N = 1,985)
Age (years)	50.5 (18.9)	50.1 (17.7)	62.0 (11.4)	63.9 (12.4)
Height (cm)	177.8 (7.0)	163.8 (6.5)	174.2 (7.0)	159.7 (6.9)
Weight (kg)	89.0 (14.9)	71.2 (15.9)	86.1 (14.4)	69.4 (15.0)
BMI (kg/m^2^)	28.9 (4.3)	27.3 (6.0)	28.3 (4.2)	27.2 (5.5)
Fat mass (kg)	23.5 (8.9)	26.9 (10.3)	22.8 (6.2)	27.7 (8.7)
Hip/FN BMD (g/cm^2^)	1.03 (0.15)	0.91 (0.14)	0.96 (0.14)	0.84 (0.16)

Note: Presented are means (SD).

**Table 2:** Information on the *SOX6* gene SNPs bivariately associated with obesity and osteoporosis phenotypes in the male subjects of our GWAS.

SNP	Position	Role	Allele^a^	MAF^b^	MAF^c^	Univariate P value			Bivariate P value	
						BMI	FM	Hip BMD	BMI-hip BMD	FM-hip BMD
*rs297325*	16346170	Intron 1	C/T	0.229	0.225	0.32	0.15	0.80	6.82×10^−7^	5.67×10^−7^
*rs4756846*	16360087	Intron 1	C/T	0.119	0.144	0.07	0.11	0.12	1.47×10^−6^	1.21×10^−6^

Note:

a The first allele represent the minor allele of each locus.

b Minor allele frequency calculated in our own Caucasian sample.

c Minor allele frequency reported for Caucasians in the public database of HapMap CEU.

The two SNPs are in the intron 1 of the *SOX6* gene. According to the analysis using the HaploView program, the two SNPs have very weak LD (*r^2^*<0.05) between each other.

To further confirm the relevance of our findings to obesity, we also performed univariate analysis of FM as well as bivariate analysis of FM-hip BMD at the two SNPs (Table 2). Again, at the univariate level, the SNPs were not found to be associated with FM at the significance level of *p* = 0.05. However, at the bivariate level, the SNPs achieved highly significant *p* values for bivariate association with FM-hip BMD (Table 2).

The SNP, *rs4756846*, also showed predictive efficacy for obesity. We stratified our male subjects in our GWAS cohort into obese and normal groups based on the diagnostic criterion for obesity (i.e., BMI≥30 kg/m^2^). In our male subjects, 146 subjects are defined as obese and the remaining 353 are normal. According to our analysis, for the SNP *rs4756846*, the non-carriers of the minor allele, C, has an odds ratio of 1.75 (*p* = 0.048) for obesity, as compared to the carriers.

These two *SOX6* SNPs ranked at the top in significance for bivariate association with BMI-hip BMD in the male subjects among all the ∼380,000 SNPs examined genome-wide. The two SNPs also ranked among the top 5 SNPs for bivariate association with FM-hip BMD in the male subjects among all the SNPs tested genome-wide. For readers' information, we list in Appendix S2 the top 5 SNPs for bivariate association with BMI-hip BMD and the top 5 SNPs for association with FM-hip BMD. Due to the top significance achieved by these two SNPs in our GWAS, our replication analyses in the FHS cohort were focused only on these two SNPs.

The two SNPs were replicated in the 1,370 male subjects of the FHS cohort. The basic characteristics of the FHS cohort are summarized in Table 1. The two SNPs, *rs297325* and *rs4756846*, achieved *p* values of 0.03 and 0.02, respectively, for bivariate association with BMI-FN BMD. In addition, the two SNPs achieved *p* values of 0.04 and 0.08, respectively, for bivariate association with FM-FN BMD. The detailed results are shown in Table 3. In the male subjects of the FHS cohort, the correlation coefficient between BMI and femoral neck (FN) BMD is 0.257 (*p*<0.001), and that between FM and FN BMD is 0.079 (*p* = 0.015).

**Table 3:** Replication signals for the *SOX6* gene SNPs in the male subjects of the FHS cohort.

SNP	Position	Role	Allele^a^	MAF^b^	MAF^c^	Univariate P value			Bivariate P value	
						BMI	FM	FN BMD	BMI-FN BMD	FM-FN BMD
*rs297325*	16346170	Intron 1	C/T	0.212	0.225	0.19	0.05	0.07	0.03	0.04
*rs4756846*	16360087	Intron 1	C/T	0.118	0.144	0.21	0.90	0.03	0.02	0.08

Note:

a The first allele represent the minor allele of each locus.

b Minor allele frequency calculated in the FHS cohort.

c Minor allele frequency reported for Caucasians in the public database of HapMap CEU.

Using Fisher's method [21], we combined the bivariate *p* values achieved in the GWAS cohort with those achieved in the FHS cohort (Table 4). Compared with *rs4756846*, the combined bivariate *p* values at the *rs297325* were more significant, which are 3.83×10^−7^ for bivariate association with BMI- BMD and 4.22×10^−7^ for bivariate association with FM- BMD.

**Table 4:** Combined bivariate *p* values of the *SOX6* gene SNPs.

SNP	GWAS bivariate P values		FHS bivariate P values		Combined bivariate P values	
	BMI-hip BMD	FM-hip BMD	BMI-FN BMD	FM-FN BMD	BMI-BMD	FM-BMD
*rs297325*	6.82×10^−7^	5.67×10^−7^	0.03	0.04	3.83×10^−7^	4.22×10^−7^
*rs4756846*	1.47×10^−6^	1.21×10^−6^	0.02	0.08	5.39×10^−7^	1.66×10^−6^

Since under univariate analysis, association of the two *SOX6* SNPs with hip BMD, BMI and FM is non-significant in both the GWAS and the FHS cohorts, we are unable to estimate if the direction of effects for the SNPs is the same in the two cohorts.

We analyzed our GWAS cohort using software Structure 2.2 [22]. When 200 randomly selected un-linked markers were used to cluster our subjects, under all the assigned values (i.e., 2, 3, and 4) for the assumed number of population strata, *k*, all the subjects were tightly clustered together, suggesting no population stratification. The results are shown in Appendix S3. We further tested the cohort for population stratification using EIGENSTRAT software [23]. Based on genome-wide SNP information, we estimated inflation factor (λ), a measure for population stratification, for each of the three traits (BMI, FM, and hip BMD) tested in this study. Ideally, for a homogenous population with no stratification the value of λ should be equal or near to 1. In our GWAS cohort, the estimated values for λ for BMI, FM, and hip BMD were 1.003, 1.007, and 1.009, respectively, which suggested no population stratification and further confirmed the results from the Structure 2.2 software.

## Discussion

With GWAS becoming a convenient and powerful tool for genetic study of common human diseases, an arising new challenge is how to utilize efficiently the vast amount of information generated in GWAS to better understand disease mechanisms. Currently, GWAS are normally performed in a univariate framework, which analyzes different phenotypes in isolation even for a single study population. Such an approach ignores the genetic correlation between and genetic co-predisposition to many human diseases, which is a common scenario in modern medicine. To address the above shortcomings, new GWAS strategies, such as bivariate association approach as adopted in the present work to study co-variation of two related disease phenotypes, are necessary. The new strategy, with more efficient use of GWAS data, may help identify pleiotropic genes underlying diseases of shared genetic susceptibility and help reveal the interconnected pathophysiological networks for a spectrum of common human diseases of major public health importance.

With the novel multivariate approach, we here performed the first genome-wide bivariate association analyses for obesity and osteoporosis. Our study identified in the male subjects *SOX6* as a potential pleiotropic gene underlying both obesity and osteoporosis. Two SNPs of the gene achieved bivariate association with BMI-hip BMD and with FM-hip BMD, with the bivariate *p* values ranking at the top among ∼380,000 SNPs tested genome-wide (Appendix S2). The bivariate association detected in our GWAS was confirmed also in the male subjects from an FHS cohort (Table 3).

In addition to the above statistical evidence, previous biological studies on the *SOX6* gene also support its dual role in both obesity and osteoporosis. *SOX6* is a member of the *SOX* gene family that encodes a group of transcription factors defined by the conserved high mobility group (HMG) DNA-binding domain [24]. As documented in the OMIM website (http://www.ncbi.nlm.nih.gov/entrez/dispomim.cgi?id=607257), the major function of *SOX6* is chondrogenesis and cartilage formation. The gene was found to be expressed during mouse chondrogenesis and to activate a chondrocyte differentiation marker, *COL2A1* [25]. Null mutations of the gene in mice caused skeletal abnormalities through influencing size and mineralization rate of endochrondral elements [26]. In respect to the gene's relevance to obesity, two recent studies identified that *SOX6* plays an important role in obesity-related insulin resistance [27], [28]. The gene was found to attenuate glucose-stimulated insulin secretion and was downregulated in the pancreatic beta-cells in hyperinsulinemic obese mice; the gene's downregulation may further stimulate beta-cell proliferation and insulin secretion. The above evidence, together with our GWAS findings, may prompt us to propose a hypothetical mechanism for co-regulation of obesity and osteoporosis, where the *SOX6* gene's effects on chondrocytes and pancreatic beta cells may play a key role. However, this mechanism is still speculative and needs extensive studies for final validation.

Bivariate association analyses as adopted in this study is a powerful approach in identifying pleiotropic genes for genetically correlated complex diseases/traits, such as obesity and osteoporosis. As shown in our statistical power analyses (Appendix S1), association analyses in a bivariate framework are more powerful than regular univariate association analyses for any of two genetically correlated traits. This difference in power between univariate and bivariate analyses is clearly reflected in our results. As shown in Table 2, although in our GWAS none of the two *SOX6* gene SNPs achieved nominally significant *p* values (*p*<0.05) for univariate association with BMI, FM or hip BMD, both SNPs achieved *p* values of 10^−6^ to 10^−7^ for bivariate association with BMI-hip BMD or FM-hip BMD. Of note is that the *SOX6* gene and its SNPs would not have been discovered for the significance to obesity and osteoporosis in our GWAS if only univariate association analyses were performed. Our study results underscore the advantage of bivariate over univariate association approaches in detecting pleiotropic genetic variants for complex diseases/traits, especially given that these variants quite often have only moderate effects to an individual phenotype/trait and hence may be insensitive to regular univariate genetic association analysis.

In this study, we intentionally restricted our selection of study phenotypes to those most important ones for obesity and osteoporosis research. For osteoporosis, we chose only “hip BMD” as the study phenotype since it is one of the most frequently measured skeletal sites for assessing osteoporosis. More importantly, hip BMD is directly relevant to risk of hip fracture, the most severe and fatal outcome of osteoporosis. Therefore, findings based on hip BMD may be clinically more important than other osteoporosis phenotypes, such as spine BMD. For obesity, we chose BMI and FM as study phenotypes due to the following reasons. The WHO proposed BMI as a simple practical measure for obesity. In epidemiological studies, BMI is also the most commonly used obesity phenotype. We chose FM as another obesity phenotype in order to corroborate findings achieved through studying BMI since BMI alone may not always be appropriate in defining obesity. For example, a very muscular soldier with only 10%–15% body fat may have a BMI>25 kg/m^2^ [29]. Therefore, our bivariate association analyses were focused only on two pairs of phenotypes, BMI-hip BMD and FM-hip BMD.

Currently, there is still no standard method to deal with the multiple testing problem in a GWAS and hence the cut-off *p* value for a significant association in a GWAS is not well defined. A genome-wide significance threshold of *p* = 4.2×10^−7^ was recently proposed by Lencz et al. [30] based on a Bayesian approach [31] (to obtain≥0.95 posterior probability of a correct inference of a genetic association) and an estimate of a total of ∼20,000 genes in the human genome. This cut-off *p* value can be used as a rough reference for the significance threshold for our study. In our GWAS, the most significant SNP, *rs297325*, achieved *p* values (6.82×10^−7^ for bivariate association with BMI-hip BMD and 5.67×10^−7^ for association with FM-hip BMD) that approach this cut-off. More importantly, this and another *SOX6* SNP, *rs4756846*, ranked at top in significance (top 2 for bivariate association with BMI-hip BMD and top 5 for association with FM-hip BMD) among the ∼380,000 SNPs tested genome-wide (see Appendix S2). Therefore, these two SNPs were selected for replication in the FHS cohort.

Population stratification and/or ethnic admixture can be an important source of spurious association in genetic association studies. However, these factors are unlikely to exist in our sample to interfere with our GWAS results. Our GWAS cohort came from an apparently homogenous US mid-west white population, living in Omaha, Nebraska and its surrounding areas. We found that the allele frequencies of the two significant SNPs in our GWAS are very similar to those reported in the typical and representative Caucasian samples used in the HapMap CEU (Table 2) and those calculated in the FHS cohort (Table 3). In the analyses using Structure 2.2 [22], all subjects used in our GWAS consistently clustered together as a single group (Appendix S3), suggesting no significant population substructure. In the analysis using EIGENSTRAT [23], the measure for population stratification, λ, for each study phenotype (BMI, FM and hip BMD) as inferred from genome-wide SNP information, was very close to 1, which also suggests no stratification in our GWAS cohort. More importantly, the association with the *SOX6* gene was replicated in an FHS cohort, a family-based study sample that is typically free from interference of population structure. For the above reasons, our association results are unlikely to be plagued by spurious associations due to population admixture/stratification.

Our findings on male-specific bivariate association with BMI-hip BMD (or FM-hip BMD) may be due to sex-specific genetic architecture for obesity and osteoporosis. Sex-specific genetic architecture has been found to be an important mechanism underlying many human common diseases and complex traits. This topic has been comprehensively reviewed in Ober et al [32]. In particular, for both obesity and osteoporosis, sex-specific genetic basis has been suggested in many studies. Sex difference in heritability of BMI was observed in a large sample containing 37,000 twin pairs from 8 countries [33]. QTL analyses in mice [34], [35] and linkage and association studies in humans [36]–[39] also revealed sex-specific genomic regions and candidate genes underlying obesity phenotypes. Sex-specific patterns were also observed in genetic studies of osteoporosis. For example, both our recent whole genome linkage scan [40] and candidate gene association study [41] identified sex-specific genomic regions and candidate genes for BMD. A large-scale meta-analysis for genome-wide linkage scans of BMD involving>11,800 subjects also suggested sex-specific genetic regulation of bone mass [42].

In summary, using a novel bivariate GWAS approach, we identified a gene, *SOX6*, which appeared to be important to co-variation of both obesity and osteoporosis risk phenotypes in male subjects. Replication of our association findings in the FHS cohort and the gene's established importance in both chondrogenesis/cartilage formation and obesity-related insulin resistance further suggests the gene's pleiotropic roles in both obesity and osteoporosis. This work also serves as a methodology exploration in GWAS, using bivariate analysis of obesity and osteoporosis phenotypes as an example. Although some evidence suggests the potential role of the *SOX6* gene in co-variation of obesity and osteoporosis phenotypes, the finding still needs to be replicated in studies of a larger-scale.

## Materials and Methods

### Subjects

#### The GWAS cohort

The study was approved by the Institutional Review Boards of Creighton University and University of Missouri–Kansas City. Signed informed-consent documents were obtained from all study participants before they entered the study. A random sample containing 1,000 unrelated Caucasians was identified and selected for this GWAS from our established and expanding genetic repertoire currently containing more than 6,000 subjects. All of the chosen subjects were US Caucasians of European origin living in Omaha, Nebraska and its surrounding areas. They were healthy subjects recruited for genetic research of common human complex traits, such as BMD and BMI. The detailed recruitment and exclusion criteria were published elsewhere [43]. Generally, subjects with chronic diseases and conditions involving vital organs (heart, lung, liver, kidney, and brain) and severe endocrinological, metabolic, and nutritional diseases were excluded from this study. In particular, for genetic study of osteoporosis, subjects with diseases and conditions that might potentially affect bone mass, structure, or metabolism were excluded. These diseases/conditions included serious metabolic diseases (diabetes, hypo- and hyper-parathyroidism, hyperthyroidism, etc.), other skeletal diseases (paget disease, osteogenesis imperfecta, rheumatoid arthritis, etc.), chronic use of drugs affecting bone metabolism (hormone replacement therapy, corticosteroid therapy, anti-convulsant drugs), malnutrition conditions (such as chronic diarrhea, chronic ulcerative colitis, etc.), and so forth. In addition, subjects taking anti-bone-resorptive or bone anabolic agents/drugs, such as bisphosphonates, were also excluded from this study. The purpose of the above exclusion procedures was to minimize the known environmental and therapeutical factors that influence or are related to the endocrine systems/factors important to development of bone mass and obesity, so that the effect sizes due to genetic factors can be enhanced in our study sample for more powerful detection of genetic variants via our study design.

BMI was calculated as body weight (in kilograms) divided by the square of height (in meters). Weight was measured in light indoor clothing without shoes, using a calibrated balance beam scale, and height was measured using a calibrated stadiometer. We also measured body fat mass (FM) using a Hologic 4500 DEXA machine (Hologic Inc., Bedford, MA) in the study subjects. The short-term reproducibility (coefficient of variation, CV) of BMI and FM measurements was on average 0.2% and 1.1%, respectively. Hip BMD values were measured also using the DEXA machine. The CV of the DXA measurements for hip BMD was 1.98%. The general relevant characteristics of the study subjects were listed in Table 1.

#### The FHS replication cohort

To replicate our GWAS findings, we used a sample from the FHS population, which contains 3,355 Caucasians, including 1,370 males and 1,985 females, from 975 families. The phenotype and genotype information of the cohort was downloaded from Framingham SHARe (SNP Health Association Resource), accessed through NCBI dbGaP (http://view.ncbi.nlm.nih.gov/dbgap). Appropriate procedures have been taken for the usage of the data, which include approval from UMKC IRB and signatures on the Data Distribution Agreement by all the UMKC investigators who have access to the data.

BMI, FM and femoral neck (FN) BMD information was available for all the study subjects according to the Framingham SHARe. The basic characteristics of the study subjects are presented in Table 1.

### Genotyping

#### GWAS cohort

Genomic DNA was extracted from whole human blood using a commercial isolation kit (Gentra systems, Minneapolis, MN, USA) following the protocols detailed in the kit. Genotyping with the Affymetrix Mapping 250k Nsp and Affymetrix Mapping 250k Sty arrays was performed using the standard protocol recommended by the manufacturer. Genotyping calls were determined from the fluorescent intensities using the DM algorithm with a 0.33 *p*-value setting [44] as well as the B-RLMM algorithm [45]. DM calls were used for quality control while the B-RLMM calls were used for all subsequent data analysis. B-RLMM clustering was performed with 94 samples per cluster.

In our GWAS genotyping experiment, following an Affymetrix guideline, we set a standard for the minimum DM call rate at 93% for a sample, considering all the SNPs in the two arrays (i.e., the 250k Nsp and 250k Sty arrays). 99% of all the subjects (i.e., 990 subjects among a total of 1,000 subjects) met this call rate standard. The remaining 10 samples that did not meet this standard however had one hybridized array passing or approaching this call rate standard (i.e., 93% of all the SNPs in the array were successfully called). Hence the genotype data in the array (with the higher call rate) for these 10 samples were also kept in the dataset for GWAS analysis. For all the 1,000 subjects, the average DM call rate reached>95%.

The final average BRLMM call rate across the entire sample reached the high level of 99.14%. However, out of the initial full-set of 500,568 SNPs, we discarded 32,961 SNPs with sample call rate<95%, another 36,965 SNPs with allele frequencies deviating from Hardy-Weinberg equilibrium (HWE) (*p*<0.001) and 51,323 SNPs with minor allele frequencies (MAF)<1%. Therefore, the final SNP set maintained in the subsequent analyses contained 379,319 SNPs, yielding an average marker spacing of ∼7.9 kb throughout the human genome.

#### FHS replication cohort

Using the FHS cohort, we performed *in silico* replication of two interesting SNPs (*rs297325* and *rs4756846*) identified in our GWAS cohort (see details for the SNPs in the Results section and in Table 2). Genotyping of the FHS cohort was performed with Affymetrix 500K mapping array plus Affymetrix 50K supplementary array. For details of the genotyping method, please refer to Framingham SHARe at NCBI dbGaP website (http://www.ncbi.nlm.nih.gov/projects/gap/cgi-bin/study.cgi?study_id=phs000007.v3.p2). Specifically, for both of the two SNPs of interest, the call rate is 99.8%. The *p* values for HWE test at the two SNPs are 0.22 for *rs297325* and 0.86 for *rs4756846*, suggesting HWE and good genotyping quality at the SNPs.

### Statistical Analyses

We compared statistical power of bivariate association analyses of two continuous traits with that of univariate association analyses of each trait separately. Under three genetic models, i.e., additive, dominant, and recessive, we performed power analyses using the GEE (Generalized Estimation Equation) Package implemented in the R environment (http://cran.r-project.org/src/contrib/Descriptions/geepack.html) for genotype-based bivariate association analyses. We used ANOVA in R and performed power analyses of genotype-based univariate association analyses. The power analyses were based on a sample size of 500 unrelated subjects (in consistence with our gender-specific GWAS analysis, where the sample size is ∼500). One thousand replicates were run in simulation to calculate the power. The detailed procedures of simulation for power calculation are presented in Appendix S1.

Bivariate GWAS analyses were performed using SAS (SAS Institute Inc., Cary, NC), where bivariate regression analyses were conducted to detect association between a SNP and two phenotypes. The analyses were based on a linear model. Denote, for an individual 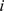 , 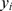 be a vector of a length of 2, coding the individual's bivariate phenotype, which can be modeled as
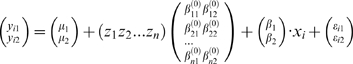
where 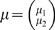 is the grand mean vector, 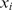 is the genotype score at the locus of interest for individual 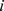 , 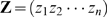 is a vector coding for covariates and may include other risk factors and confounding factors, 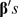 represent the corresponding effects of covariates or the SNP under test, and 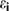 is the random error vector. We tested the alternative hypothesis by comparing the likelihood of the model under the null hypothesis (SNP effects are restricted to 0) with that under the alternative hypothesis (the SNP effects are not 0). The likelihood ratio can convert to an *F*-statistic, which follows an *F*-distribution under the null hypothesis. The bivariate *p* value was then calculated based on the *F*-statistic.

We have recently published two papers that used approaches similar to that as shown above for bivariate association analyses [46], [47].

In the FHS cohort, a family-based sample, bivariate association analyses were performed using FBAT-GEE implemented in FBAT (ver. 2.02) (http://biosun1.harvard.edu/~fbat/fbat.htm) [48]. FBAT-GEE generalizes univariate family-based association analyses to multivariate scenarios. It can produce a *_X_* ^2^ *FBAT-GEE* statistic, which follows a chi-square distribution and its degrees of freedom is the number of phenotypes tested. The bivariate *p* value was then calculated based on the statistic.

For comparison purpose, we also calculated univariate association with each tested phenotype using SAS (SAS Institute Inc., Cary, NC) in our GWAS cohort and using FBAT in the FHS cohort. For analysis using SAS in our GWAS cohort, genotypic association analysis was performed under the linear regression framework, where genotype was treated as the independent variable and the study phenotype (such as BMI) as the dependent variable, and the phenotype was modeled as a linear function of alternative genotypes at a certain SNP. For analysis using FBAT in the FHS cohort, association analysis was performed by correlating transmission of parental genotype to offspring with a phenotype.

Additive genetic model was applied in both univariate and bivariate association analyses.

In all the above association analyses, age, age^2^ and sex were included as covariates to adjust the study phenotypes. In the gender specific association analyses, only age and age^2^ were used as the covariates.

The linkage disequilibrium (LD) between interesting SNPs was analyzed using the Haploview program [49] (http://www.broad.mit.edu/mpg/haploview/) and the most recent SNP genotype data (HapMap Data Rel 23a/phaseII Mar 08, on NCBI B36 assembly, dbSNP b126) from HapMap (www.hapmap.org).

To quantify the overall evidence of association achieved in our GWAS and in the FHS replication cohort, Fisher's method [21] was used to combine the individual *p* values achieved in our GWAS and FHS cohorts. The method, also known as Fisher's combined probability test, is a meta-analysis technique for combining the results from independent statistical tests that have the same overall null hypothesis (*H_0_*) [21]. The method combines *p* values from different studies into one test statistic that has a chi-square distribution using the formula 
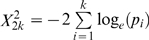
. The *p* value for the *X* ^2^ statistic can be extrapolated from a chi-square table using 2*k* “degree of freedom”, where *k* is the number of tests being combined.

To detect population stratification that may lead to spurious association results, we used the software Structure 2.2 (http://pritch.bsd.uchicago.edu/software.html) and EIGENSTRAT software (http://genepath.med.harvard.edu/~reich/EIGENSTRAT.htm) to investigate the potential substructure/stratification of our sample. The Structure 2.2 program uses a Markov chain Monte Carlo (MCMC) algorithm to cluster individuals into different cryptic sub-populations on the basis of multi-locus genotype data [22]. Using the software, we performed independent analyses under three assumed numbers of population strata, *k* = 2, 3, and 4, respectively, using 200 un-linked markers randomly selected genome-wide. To confirm the results achieved through Structure 2.2, we further tested population stratification in our sample using EIGENSTRAT software that uses principal component analysis approach to model ancestral differences between cases and controls [23].

## Supporting Information

Appendix S1 (0.12 MB DOC)

Appendix S2 (0.05 MB DOC)

Appendix S3 (0.07 MB DOC)
