# Supplementary material for: Powerful Bivariate Genome-Wide Association Analyses Suggest the SOX6 Gene Influencing Both Obesity and Osteoporosis Phenotypes in Males
Source: PLoS One. 2009 Aug 28;4(8):e6827. doi: 10.1371/journal.pone.0006827 (PMC2730014; doi:10.1371/journal.pone.0006827)

**Appendix S1. Comparison of Statistical Power for Bivariate vs. Univariate Association Analyses**

1. **Data simulation:**

One causal SNP (QTL) was randomly generated, of which, the MAF was set at 0.3. The SNP effect (QTL heritability, *h^2^*) equals to$\sigma_{q}^{2}/(\sigma_{q}^{2}+\sigma_{e}^{2})$, where$\sigma_{q}^{2}$ is the genetic variance of the SNP and$\sigma_{e}^{2}$, the variance of the environment effect, was set at 1. We further decomposed $\sigma_{q}^{2}$into the additive genetic variance,$\sigma_{a}^{2}$, and the dominant genetic variance, $\sigma_{d}^{2}$, such that $\sigma_{q}^{2}=\sigma_{a}^{2}+\sigma_{d}^{2}$. $\sigma_{a}^{2}$ equals to 2*pqa*^2^, where *a* is the additive effect. $\sigma_{d}^{2}$equals to (2*pqd*)^2^, where *d* is the dominant effect and we set $d=k*a$ and $k=0,1,-1$ for additive, dominant and recessive models, respectively.


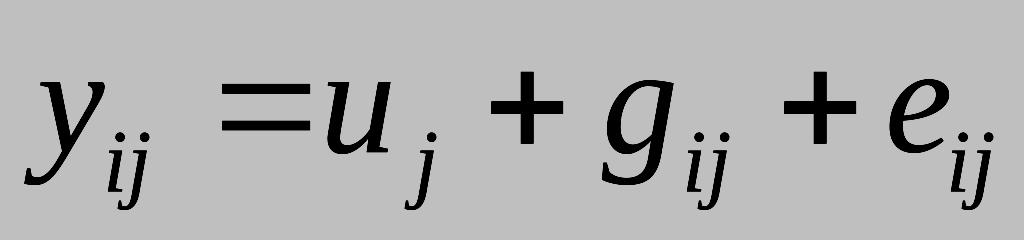
Two quantitative traits were simulated by the following formula,

where $u_{j}$ is the mean of the sample (we set it as 5.0), $g_{ij}$ is the genetic effect of the SNP, $e_{ij}$ is the environmental residuals.

We assume that the causal SNP (QTL) affects the two traits simultaneously, i.e., the SNP has the same degree of genetic effect on both traits. Thus the phenotypic values of the two traits of an individual *i* can be generated conditional on the genotype at the QTL via a bivariate normal distribution with the mean $\left(\begin{matrix} u_{1}+g_{i1} & u_{2}+g_{i2} \end{matrix}\right)^{'}$ and variance matrix $\left(\begin{matrix} 1 & r \\ r & 1 \end{matrix}\right)$. Here *r* is the environmental correlation coefficient and we set *r* = 0.5 in our simulations**.**

The significance level for the bivariate genetic association analysis was set as *p*=0.05, and the significance level for univariate genetic association analysis was set as min(*p*1, *p*2)=0.025, to adjust for multiple testing using Bonferroni correction, where *p*1 and *p*2 are the *p* values for trait 1 and trait 2, respectively, in univariate analysis.

1. **Parameter setting:**

The following table summarizes the detailed parameters we set for simulation under each genetic model.

| MAF | Additive model | | | |  | Dominant model | | | |  | Recessive model | | | |
| --- | --- | --- | --- | --- | --- | --- | --- | --- | --- | --- | --- | --- | --- | --- |
|  | QTL *h^2^* | *a* | *k* | *σ_e_^2^* |  | QTL *h^2^* | *a* | *k* | *σ_e_^2^* |  | QTL *h^2^* | *a* | *k* | *σ_e_^2^* |
| 0.3 | 0.005 | 0.1094 | 0 | 1 |  | 0.02 | 0.1850 | 1 | 1 |  | 0.003 | 0.0710 | -1 | 1 |
|  | 0.010 | 0.1551 | 0 | 1 |  | 0.04 | 0.2643 | 1 | 1 |  | 0.006 | 0.1006 | -1 | 1 |
|  | 0.015 | 0.1904 | 0 | 1 |  | 0.06 | 0.3271 | 1 | 1 |  | 0.009 | 0.1234 | -1 | 1 |
|  | 0.020 | 0.2204 | 0 | 1 |  | 0.08 | 0.3818 | 1 | 1 |  | 0.012 | 0.1427 | -1 | 1 |
|  | 0.025 | 0.2471 | 0 | 1 |  | 0.10 | 0.4316 | 1 | 1 |  | 0.015 | 0.1598 | -1 | 1 |

1. **Results:**

The following figures compare statistical power between bivariate and univariate association analyses under three different genetic models, i.e., additive, dominant, and recessive, and five different SNP effect sizes.

**
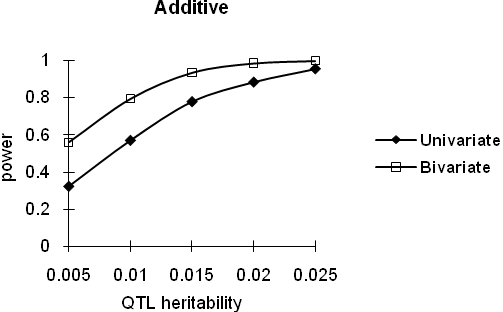
**

**
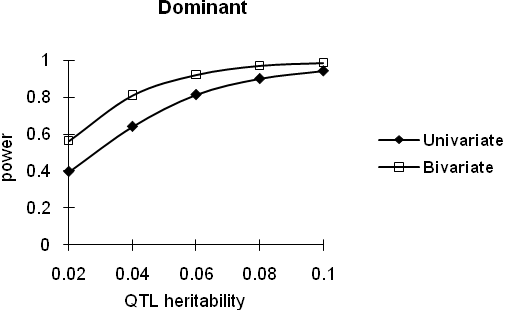
**


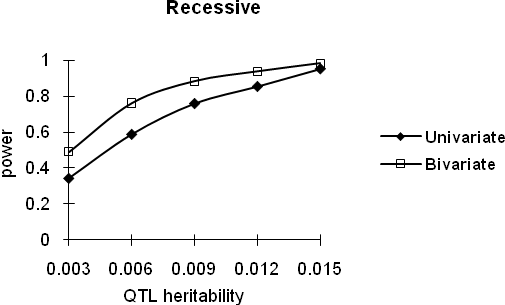

Supplement: Appendix S1 — (0.12 MB DOC) [file pone.0006827.s001.doc]
