# Supplementary material for: Powerful Bivariate Genome-Wide Association Analyses Suggest the SOX6 Gene Influencing Both Obesity and Osteoporosis Phenotypes in Males
Source: PLoS One. 2009 Aug 28;4(8):e6827. doi: 10.1371/journal.pone.0006827 (PMC2730014; doi:10.1371/journal.pone.0006827)
Supplement: Appendix S2 — (0.05 MB DOC) [file pone.0006827.s002.doc]

**Appendix S2. The Top 5 SNPs Identified in the Bivariate GWAS in Males**

| Ranking | SNP | Gene | Univariate P value | | Bivariate P value |
| --- | --- | --- | --- | --- | --- |
|  |  |  | BMI | Hip BMD | BMI-hip BMD |
| 1 | **rs297325** | SOX6 | 0.32 | 0.80 | 6.82×10^-7^ |
| 2 | **rs4756846** | SOX6 | 0.07 | 0.12 | 1.47×10^-6^ |
| 3 | rs16933105 | SOX6 | 0.37 | 0.57 | 2.51×10^-6^ |
| 4 | rs556043 | PIGZ | 0.48 | 3.62×10^-4^ | 3.68×10^-6^ |
| 5 | rs11101474 | unknown | 0.97 | 0.77 | 4.19×10^-6^ |

| Ranking | SNP | Gene | Univariate P value | | Bivariate P value |
| --- | --- | --- | --- | --- | --- |
|  |  |  | FM | Hip BMD | FM-hip BMD |
| 1 | rs556043 | PIGZ | 0.044 | 3.62×10^-4^ | 3.98×10^-7^ |
| 2 | **rs297325** | SOX6 | 0.15 | 0.80 | 5.67×10^-7^ |
| 3 | rs7515041 | unknown | 8.24×10^-4^ | 0.015 | 9.82×10^-7^ |
| 4 | rs17032192 | NCK2 | 5.81×10^-3^ | 2.95×10^-5^ | 1.09×10^-6^ |
| 5 | **rs4756846** | SOX6 | 0.11 | 0.12 | 1.21×10^-6^ |

Note:

1. The SNPs are ranked in significance for bivariate association with BMI-hip BMD or FM-hip BMD among all the ~380,000 SNPs tested genome-wide.
2. The two SNPs bolded are the SNPs selected for replication in the FHS cohort.
