# Supplementary material for: Powerful Bivariate Genome-Wide Association Analyses Suggest the SOX6 Gene Influencing Both Obesity and Osteoporosis Phenotypes in Males
Source: PLoS One. 2009 Aug 28;4(8):e6827. doi: 10.1371/journal.pone.0006827 (PMC2730014; doi:10.1371/journal.pone.0006827)
Supplement: Appendix S3 — (0.07 MB DOC) [file pone.0006827.s003.doc]

**Appendix S3. Results of Analyses of Potential Sample Stratification**


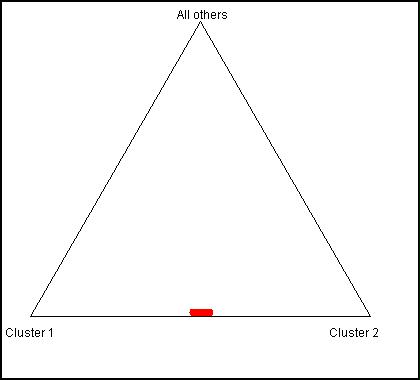


**k = 2**


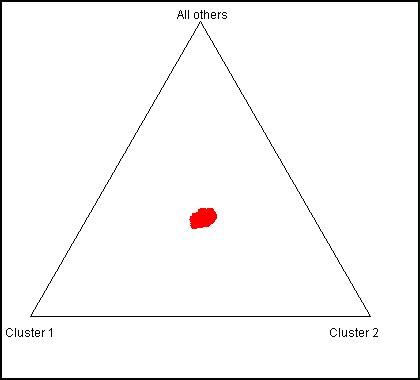


**k = 3**


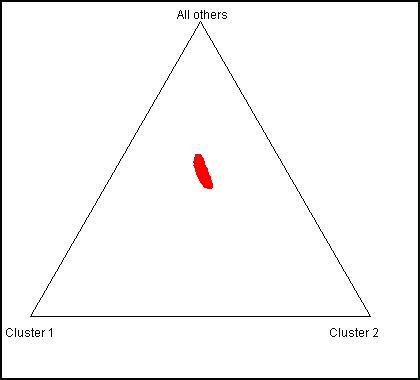


**k = 4**

Note: As shown is the output of the software Structure 2.2, which clustered our study subjects using 200 randomly selected unlinked markers under three assumed numbers of population strata, k = 2, 3, 4.
